# Robots for Foreign Language Learning: Speaking Style Influences Student Performance

**DOI:** 10.3389/frobt.2021.680509

**Published:** 2021-09-03

**Authors:** Kerstin Fischer, Oliver Niebuhr, Maria Alm

**Affiliations:** ^1^ Department of Design and Communication, University of Southern Denmark, Sonderborg, Denmark; ^2^ Centre for Industrial Electronics, University of Southern Denmark, Sonderborg, Denmark

**Keywords:** charisma, social robots, language learning, intonation, speaking style

## Abstract

Much previous research suggests that teachers’ individual characteristics may affect students’ performance; however, which factors are particularly helpful is as yet unclear and methodologically very difficult to assess. In this paper, we study the effects of robots’ speaking styles when instructing students on a task. 40 participants saw a brief video in which a robot presented its instructions either in a charismatic or a not so charismatic speaking style. Participants’ task was then to produce foreign language sentences on the basis of visualizations of the prosodic properties of these sentences. A subsequent analysis of participants’ productions shows that language learners’ performance was significantly better when the robot had delivered its instructions in a charismatic voice. The results suggest not only that a charismatic speaking style may be crucial for teachers in general and hence one of the factors causing the interpersonal variation between teachers, but also that students can benefit from instructions by robots delivered in a charismatic speaking style.

## Introduction

With robots becoming more prevalent in our daily lives and possibly our classrooms (e.g., Leyzberg et al., 2012), the question to what extent robots can facilitate learning and teaching has gained some attention (cf. Belpaeme et al., 2018 for an overview). However, so far, the use of robots in pedagogical settings has not only yielded positive results; especially in the area of language learning, i.e., in learning to interact in a foreign language, previous studies have been rather discouraging. For instance, Rosenthal-von der Pütten et al. (2016) find no positive effect of using robots for language learning, and (Jiyoung and Jeonghye, 2015) find that because of the reliance on synthesized speech, robots are not useful to teach students native-like speaking competence.

That previous work has not only found robots to be successful teachers or tutors may thus be due to the way robots speak; speech is not only the prevalent medium of communication in the classroom and the target of the language learning process, but it also conveys a lot of information about the speaker, like, for instance, the speaker’s gender, geographic origin, social class and speaker personality (Sutton et al., 2019). Of these, especially teacher personality has been shown to influence students’ success (e.g., Lee et al., 2013). Research on teaching has long noted that individual teacher characteristics may play a role in pedagogy, and scholars have tried to identify what aspects of teacher personalities may have an effect on the learning process. Suspected factors include teacher’s empathy, organization, adaptability, fostering of community, autonomy and enthusiasm (Klassen et al., 2018), and occasionally also charisma is noted as a potential factor (Towler et al., 2014; Lin and Huang 2017). That the way of speaking can have an effect has been suspected especially by experts on charismatic speech (e.g., Rosenberg and Hirschberg 2009).

However, there is no systematic investigation of the impact of the teacher’s speaking styles on students’ performance. There are at least three reasons for this: The first, methodological, reason is that very few speakers can manipulate their speaking styles to produce a charismatic speaking style in one situation and not in another, thus making the controlled investigation of speaking style difficult. The second reason is that for a long time, it has not been entirely clear what exactly makes a speaking style charismatic. The third reason is that a potential relationship between a charismatic speaking style and student performance does not matter if a charismatic speaking style cannot be taught to teachers, which is assumed by many (but see Abelin 2018).

In the current study, we circumvent the first problem by using robots as teachers. Robots, in contrast to people, can be manipulated at will, and they can produce identical output in comparable ways as often as necessary to all participants alike. Robots are therefore excellent tools for the study of effects of certain linguistic behaviors, such as speaking styles (cf. also Andrist et al., 2013, 2015; Fischer and Niebuhr 2020). In particular, robots’ speech can be presynthesized and then manipulated by a prosody expert in order to match a particular speaking style. As for the second problem area, recent research has provided evidence for a short list of factors that contribute to charismatic speech (Niebuhr et al., 2016; Berger et al., 2017). In our own previous work, we demonstrated successfully that the speech features identified in the model make robot speech more persuasive (Fischer et al., 2020). Regarding the third problem area, robots can again provide the solution: Irrespective of whether a charismatic speaking style can or cannot be taught, if we have robots deliver the instructions, we only need to know how to generate charismatic robot utterances. By using robots to investigate the effects of teachers’ speaking styles on student performance we furthermore shed light on how robots themselves can be employed to facilitate learning.

Consequently, in the current study, we focus on the characteristics of the instructions delivered by robots in a foreign language teaching context. In particular, we show that a more charismatic presentation of the task increases the correctness of students’ performance significantly. In our study, the students’ task is to interpret visualizations of the prosodic realization of questions in English, which generally constitutes a problem for them. A robot introduces the participants to the task, either using a charismatic speaking style or in a speaking style that is less charismatic. The results show that students who heard the charismatic robot produced significantly better results than students who heard the introduction by the less charismatic robot. Thus, the degree to which a teacher speaks charismatically can influence students’ performance.

## Previous Work

Previous work concerns the role of teachers’ charisma in language learning situations and especially on the effects of speaking styles on learning, as well as the roles of robots in language teaching.

### Teacher Characteristics and Charismatic Speech

A review of previous work by Qardaku (2019) aims to narrow down what it means for teachers to be charismatic. Her analysis suggests that charismatic, and hence inspirational, teachers have to be experts, transmit enthusiasm for their topic and cultivate positive relationships with their students, reflect on their practices, and make learning meaningful to the students. None of these characteristics relates to the teacher’s speaking style. Also other work on charisma in teaching leaves out the verbal dimension; for example, Nissim and Simon (2019) try to identify the characteristics that teachers and teacher-leaders have in common and find that charisma is expected of a teacher-leader, and certain personality characteristics are expected of good leaders and teacher-leaders to the same extent, but that the good teacher-leader should also be able to empower students - somewhat in line with Qardaku’s (2019) suggestions. Similarly, Lin and Huang (2016) take it to be uncontroversial that a charismatic teacher is characterized by “knowledge, character, humour and teaching method,” again without any reference to speaking style. Concerning these four characteristics, they find that they are positively related to interest in a subject. In a follow-up study, Lin and Huang (2017) find that the same features also influence students’ attitude to calculus learning.

Towler et al. (2014) study the effects of what they consider charismatic content on the evaluation of the trainer and on recall and transfer immediately after the presentation and a week later. Their manipulations concern the articulation of a higher vision, positive emotional expression, emphasis of the importance of the contents, storytelling, use of metaphors, raising of expectations and encouraging of innovative thinking and the provision of encouragement and support. Students listened to a 15 min course on statistics software either with or without the charismatic features. The results show better evaluations of the charismatic teacher as well as better retention and transfer a week later. It is however unclear to what extent the trainer also used “appropriate vocal intonation,” which the authors also consider to constitute a trait of charismatic trainers.

We can conclude that as yet, there is no systematic investigation of the relationship between speaking style and student performance, and thus we don’t know the impact of a charismatic speaking style on teaching.

### Robots as Tutors in Language Learning

Robots have been found to be generally engaging for students, and to have a positive influence even on their performance (e.g., Baxter et al., 2017; De Haas et al., 2020). However, robots can play various different roles in language learning situations. Belpaeme et al. (2018) provide an overview of current work on robots as language tutors (compared to as peers, as in Baxter et al.’s study) and conclude that the effects reported so far are rather small. Similarly, in a study in which the robot served as a teacher, Rosenthal-von der Pütten et al. (2016) find no advantage of an embodied robot over a computer simulation and in general neither application led students to improve their language skills. Also a large-scale study by Vogt et al. (2019) with 194 children yielded no advantages of a robot over a tablet, and the iconic gestures the robot was using (for instance, for “add”, “behind” or “running”) had no impact on children’s word learning performance. Here the robot taught 5–6 year old children English words for already known concepts. In the control condition, children sang songs together with the experimenters and were not exposed to the English words at all. Another study by De Haas et al. (2020) finds that the variability of the feedback the robot provided in an animal name learning game had an effect on children’s engagement in the game, but the feedback itself did not affect their learning gain, which was small but present in all conditions.

Rosenthal-von der Pütten et al. (2016) also investigated whether synthesized speech constitutes a problem for the language learners. They first synthesized the robot utterances, then had a human speaker speak them with similar speech characteristics in order to make the stimuli as comparable as possible. They find no differences in participants’ self-reported experiences, on alignment with syntactic and lexical features of the robot’s utterances and students’ learning gains. Thus, the synthesized utterances performed no worse than the human utterances. In contrast, In and Han (2015) found the range in speech melody in synthesized speech to be much lower than native speaker utterances, which they found to have negative effects on language learners who even adjusted their own utterances in the wrong way. The authors conclude that because of problems in speech melody, synthesized utterances are not useful for foreign language learning. By asking the human speaker to imitate the speech melody to create the stimuli, the authors in Rosenthal-von der Pütten et al.’s study may thus have eliminated the difference between synthesized and natural stimuli that In and Han (2015) identified to be most important. Thus, the fact that Rosenthal-von der Pütten et al. (2016) did not find differences between synthesized and natural speech may be related to the fact that the natural speech did not exhibit natural speech melodies.

Kory-Westlund and Breazeal (2019) investigate the role of the language level a robotic peer uses on four-six year old children’s vocabulary learning. They had a robot show a depicted scene on a tablet and tell a short story and then ask the child to tell a story about that scene, after which the robot told a story about a new scene and asked the child again to tell their own story. In the second round, the robot’s language level either did, or did not, match the child’s language competence level. The two language versions differed in syntax (simple main clauses compared to complex sentences comprising main and subclauses) and more or less complex vocabulary (for instance, basic level versus more specific general language terms). Children came in eight times to play with the robot. Children’s vocabulary scores increased more in the condition in which the robot’s language matched the child’s such that, on average, children in the matched condition picked up almost seven new words, compared to 2.5 in the unmatched condition. These results suggest that the robot’s language choices may have an effect on the amount of learning.

To sum up, while the use of robots for language learning is promising and robots have been found to increase children’s engagement and interest (at least for a certain amount of time, cf., for instance, Vogt et al., 2019), especially robots as language tutors have not been found to be very effective, and even to be counter-effective in the teaching of native-like speech melodies. However, with robots in other roles than tutors (e.g. in Baxter et al.’s and Kory-Westlund and Breazeal’s studies), it seems that speaking style might have an impact. In the current study, we therefore address whether the speaking style of robotic teachers can impact students’ performance.

## Methods

In the following, we present a study in which language learners are instructed by a robot who introduces them to the task and the experiment using either a very charismatic or a not so charismatic speaking style. The charismatic speaking style is based on the speech characteristics of Steve Jobs, whereas the other one uses the speech characteristics associated with the speech of Mark Zuckerberg. The two styles have been shown to create different pragmatic effects (e.g., Fischer et al., 2020). The learners are then asked to produce correct interpretations of three questions in English whose intonation contours and stress patterns are noted down in a prosodic notation system (cf. Fischer et al. submitted). Thus, the independent variable in this experiment is the robot’s speaking style, and the dependent variables are the errors the participants make when carrying out the task instructed. The focus of the experiment is therefore on the effect of speaking style on students’ performance.

### Stimuli

The stimuli were created by synthesizing the robot’s instructions using the male voice of a free text-to-speech system, and then manipulating them to match the speech characteristics of Steve Jobs and Mark Zuckerberg. The melodic features investigated are those that have previously been found to be related to persuasiveness and positive character traits like enthusiasm, passion, charm, and convincingness in analyses of advertisements and politicians’ speeches (Gelinas-Chebat et al., 1996; Rosenberg & Hirschberg 2005, 2009; Biadsy et al., 2007; Nienhuis 2009; Pejčić 2014; Bosker 2021). The acoustic-melodic analysis of various public speakers in Niebuhr et al. (2016) revealed that Steve Jobs’ speech features mark one end of the persuasion dimensions whereas Mark Zuckerberg’s speech characteristics mark the opposing end of the spectrum among those public speakers investigated. This juxtaposition allowed Niebuhr et al. (2016) to identify potentially influential charismatic speech features, and several studies (Berger et al., 2017) confirm that those speech characteristics are indeed related to charisma, even when used by robots (Fischer et al., 2020).

For the manipulations, we used the PSOLA pitch and duration manipulation functions available in *praat* (Boersma 2001). Changes in acoustic energy were made using Audacity (www.audacityteam.org/). The manipulations resulted in two different versions of the same instructions, one time with the speech characteristics identified for Steve Jobs and another one with those identified for Mark Zuckerberg.

Table 1 provides an overview of the acoustic-melodic parameters manipulated. In general, these features concern the pitch level (measured in semitones relative to a male baseline of 100 Hz, st), i.e., how high or low the fundemental frequency is; the pitch range (measured in semitones, st), i.e., how far up and how far low a given voice moves; the acoustic-energy level (RMS, measured in decibel, dB, and normalized to the dB level of a frequent reference word (“so”) in the two speaker’s speeches), i.e., how loud the voice is; the speaking rate (measured in syllables per second, syl/s, excluding pauses), i.e., how fast or slow the speech is; the emphatic accent frequency (measured in counts per minute, cpm), i.e., how often a speaker adds expressive accentuation to stressed words; the hesitation frequency (measured in counts per minute, cpm), i.e., how often a speaker uses *uh* and *um*; the duration of silent pauses (measured in deciseconds, ds), i.e., how long the silence lasts; and the frequency of high-pitched accents (measured in counts per minute, cpm). Note that all values in Table 1 refer to mean values and, thus, to the two speakers’ speeches as a whole. Accordingly, we also took them as target values for the robot’s utterances as a whole.

**TABLE 1:** The acoustic features manipulated.

Acoustic speech feature	Steve Jobs	Mark Zuckerberg
Mean pitch level relative to 100 Hz (st)	8.8	5.4
Mean pitch range (st)	22.9	12.1
Mean acoustic-energy level normalized relative to all instances of “so” (dB)	−3.2	−5
Mean speaking rate (syl/s)	4.4	5.9
Emphatic accent frequency (cpm)	8.4	1.6
Mean silent pause duration (ms)	200	500
Frequency of high-pitched accents (cpm)	17.2	13.8

When applying these measurements to the robot’s utterances, we focused on those acoustic features of the speech signal that were found relevant in the comparison between the different speaking styles of Steve Jobs and Mark Zuckerberg, but we also took into account that the acoustic manipulation would still produce naturally sounding and comparable stimuli. For example, while voice quality is potentially relevant in the perception of the speaker’s personality (cf., for instance, Signorello and Demolin 2013), it is difficult to manipulate voice quality, given state-of-the-art resynthesis tools. We therefore restricted the manipulation to the features listed in Table 1, which have also been shown to be effective in Fischer et al. (2020), in which the two speaking styles led to different behavioral effects.

During the creation of the robot speech stimuli, the manipulation procedure was conducted iteratively by adjusting each parameter successively. This is necessary because a resynthesis is required after each manipulation before the effect of the manipulation can be evaluated. Thus, the manipulations were applied individually and in as many iterations as necessary to achieve the values described in Table 1.

We deemed a manipulation check of our stimuli unnecessary because of extensive previous work that has shown that the two manipulations have significant effects on the speaker’s perceived charisma. Specifically, Berger et al. (2017) and Niebuhr (2021b) show in detail that the two speaking styles employed, inspired by Steve Jobs and Mark Zuckerberg respectively, have significantly different effects on the extent to which they are perceived as charismatic. Michalsky and Niebuhr (2019) and Fischer et al. (2020a) furthermore show that the same effect occurs with artificial speakers, like in-car navigation systems and a range of different robots, including Keepons; for instance, in the study involving the Keepon robots, Fischer et al. (2020a) find that the robots that use the speaking style inspired by Steve Jobs to be significantly more passionate, enthusiastic and charming, as well as significantly less boring. We can thus safely assume that the stimuli used in this experiment will yield similar interpretations of the robots as charismatic or not.

The audio files with the instructions were then combined with a video in which a Keepon robot moved slightly as if in coordination with speaking. The text the robot produced was:

*Hello, we are the Keepons! Thanks for taking the time for this little exercise! We want to teach you how to ask questions in English with the right speech melody. First, my kind human assistant will ask you to fill out a consent form. After that, my human assistant will show you three questions with representations of the speech melody and ask you to record these questions. That’s all! Thank you so much already!*

The actual task participants had to fulfill was to produce three questions in English with the appropriate speech melodies. Even though English and Danish are both Germanic languages, the intonation patterns of the two languages are quite different. In particular, in our previous studies (Niebuhr et al., 2017; Fischer et al. submitted), it turned out that native speakers of Danish, who are the participants in our study, have great problems with the production of the rising final tonal gesture in English questions because there is no such rise in questions in Danish; instead, the signaling of speech acts (statements vs. questions) is indicated by the declination of an utterance as a whole (Grønnum 2007: 98). Thus, producing final rises in English questions constitutes a challenge for Danish learners of English as a foreign language. Consequently, we expected our participants to have problems with the task (cf. also Niebuhr et al., 2017).

Participants’ task consisted in reading three questions out loud based on a visualization of the speech melody and stress placement of three native-speaker utterances annotated according to ToBI by Hedberg et al. (2017): (10). The examples are thus based on authentic American English questions, more precisely on examples of questions with a low-rise nuclear contour, which Hedberg et al. (2017) find to be the unmarked nuclear contour for *yes-no* questions in their corpus study of American English. We re-interpreted the ToBI notation into drawn intonation contours complemented with stress marking for the prominent syllables (cf. Ladd, 2014). We only made one small adjustment to the third example (Do you still work for a veterinarian) by removing the third, contrastive accent in the sentence because we deemed such a contrastive accent to be confusing without a supporting context. The visualization technique of the intended prosodic realization of these questions had been developed in several experiments and had proven to yield the best results, compared to six other common notation systems (Fischer et al. submitted). Figure 2 shows the visualizations presented to the students.

The task, to read English sentences based on a visualization of their prosodic realization, is quite difficult for foreign language learners because we do not normally produce intonation contours voluntarily, and because the original English contours feel strange for the native speaker of Danish, where rises in the final intonation contour do not occur. Thus, the task is sufficiently challenging so that ceiling effects are prevented and students are quite likely to fail to some extent. At the same time, it is a realistic and informative task, because students learn to produce English questions in the appropriate way (otherwise risking negative inferences about their personalities, since the transfer of intonation contours from the native to the target language usually leads to unwanted conclusions about a speaker’s character cf. Fischer and Niebuhr (2020) concerning the effects of transferring Danish contours into a language in which a final rising contour is expected]. And finally, even though the role of the robot is not to explain how questions in English are to be produced, the task is relatively typical of teaching situations in which teachers provide students with access to resources, like pronunciation dictionaries, that allow them to improve their productions, or ask them to fill out exercising sheets.

### Procedure

Forty participants (21 female, 19 male), twenty in each condition, were recruited by three student assistants by approaching them while they were sitting in the common spaces at three campuses of a large Danish university. Participants were all students from a broad range of disciplines and both undergraduates and graduates. Given the prominent role of English in Danish society in general (for instance, panel discussions at prime time may be held in English on Danish TV if international guests are involved), the early introduction of English in school (most often as the first foreign language), and the ubiquity of English at the university in particular, where many courses are taught in English, we can understand all Danish students to be learners of English as a foreign language at an advanced level. The participants were between 18 and 55 years old, with an average age of 26 and a median age of 24.

**FIGURE 1:**
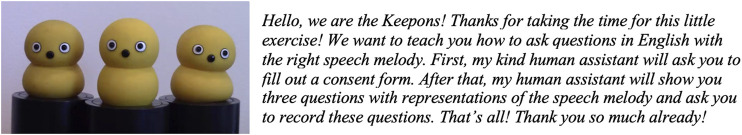
The Keepon robots used and the text that the robot in the middle presented.

**FIGURE 2:**
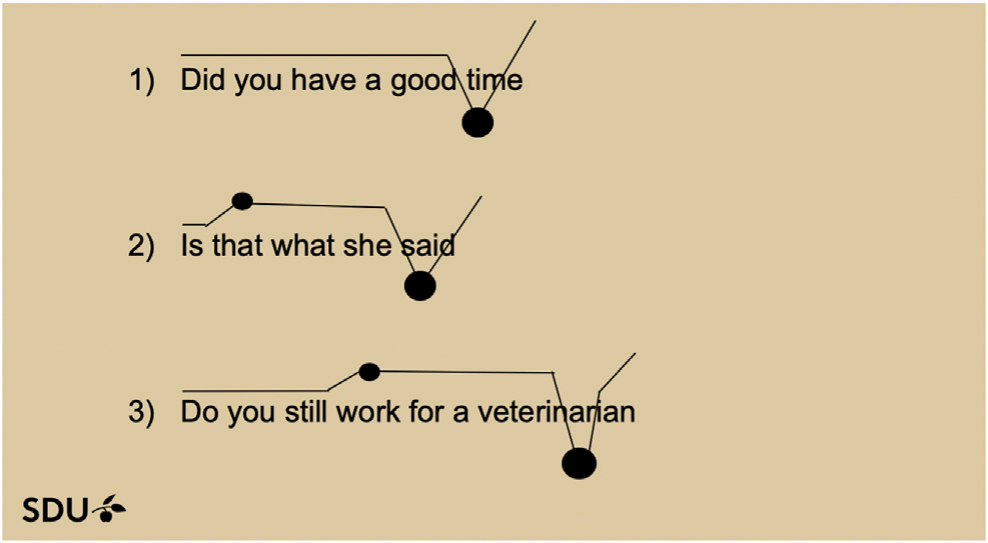
The three questions to be produced.

Participants were given a tablet that played a Powerpoint presentation, where on the first slide they saw a video in which a Keepon robot, the middle robot in a group of three robots (see Figure 1), welcomed participants to the experiment and briefly explained the procedure. On the next slide, the students found a link to a consent form, informing them about their right to withdraw from the experiment at any time, and asking them for the permission to record their data, to analyze the data and to publish their data, for instance, at a conference, in separate questions. Then, participants were presented with the visualizations of three English questions (see Figure 2), which they were asked to read out loud with the intonation contour and stress pattern visualized. Participants were allowed to practice as often as they like and then provided with an external digital recording device to record their realizations of the three questions.

### Data Analysis

In the data analysis, the participants’ productions were first annotated using the Kiel Intonation Model (KIM, Kohler 1997; Niebuhr 2021a). The KIM is a phonological intonation model, which analyses utterance intonation in terms of rises, falls and combinations of rises and falls, such as peaks and valleys, on an auditory basis; that is, the analysis is carried out by a prosody expert, in our case, an expert with more than 12 years of prosodic annotation experience. In addition to describing the main intonational movements of the speech melody, the annotation based on KIM also identifies stress placement. For instance, regarding our target questions displayed in Figure 2, the learners’ task is to place the stress on those syllables indicated by the dots in the visualization. The KIM allows the analysis of the placement of the stress, as well as of the right pitch movement. Accordingly, the annotation proceeds in two steps: The first step is to identify pitch-accented words and to distinguish between weak, normal and emphatic prominence levels (cf. Niebuhr et al., 2015; Baumann et al., 2016). The second step is to determine the main melodic movements connected to the stressed words. Based on the annotations of the participants’ realizations of the three questions, we analyzed the number and kinds of errors they made. We distinguish between: 1) pitch-timing errors, i.e., errors that occur if the sentence-accent realized by the participant shows a wrong timing (or f0-peak alignment) in relation to the lexically stressed syllable (Niebuhr 2013); 2) pitch-scaling errors, which describe instances in which the pitch movement is different, for instance, if a participant produces a falling contour when a rising contour is indicated (cf. Ladd, 2014); and 3) stress-level errors, i.e., errors that occur if stress is placed on another syllable than indicated. The analysis thus allows us not only to identify the extent to which students’ productions are correct, but also what kinds of mistakes they make with regard to the annotation. We are thus interested in how well our participants were able to pronounce the questions as represented by the visualizations. We did not have a panel of native speakers judging the questions in this study because the focus here is not on second-language competence in general, but on the effectiveness of the visualizations in combination with different instructor speaking styles. When we talk about errors, we mean pronunciation errors in relation to the visualizations.

## Results

The results show that students who heard the introduction by the robot whose speech was manipulated to match the speech profile of Steve Jobs performed significantly better than those students who heard the introduction from the robot whose speech characteristics matched those of Mark Zuckerberg. Given that each participant produced three questions, there are 60 opportunities for each error type to occur in each condition.

**FIGURE 3:**
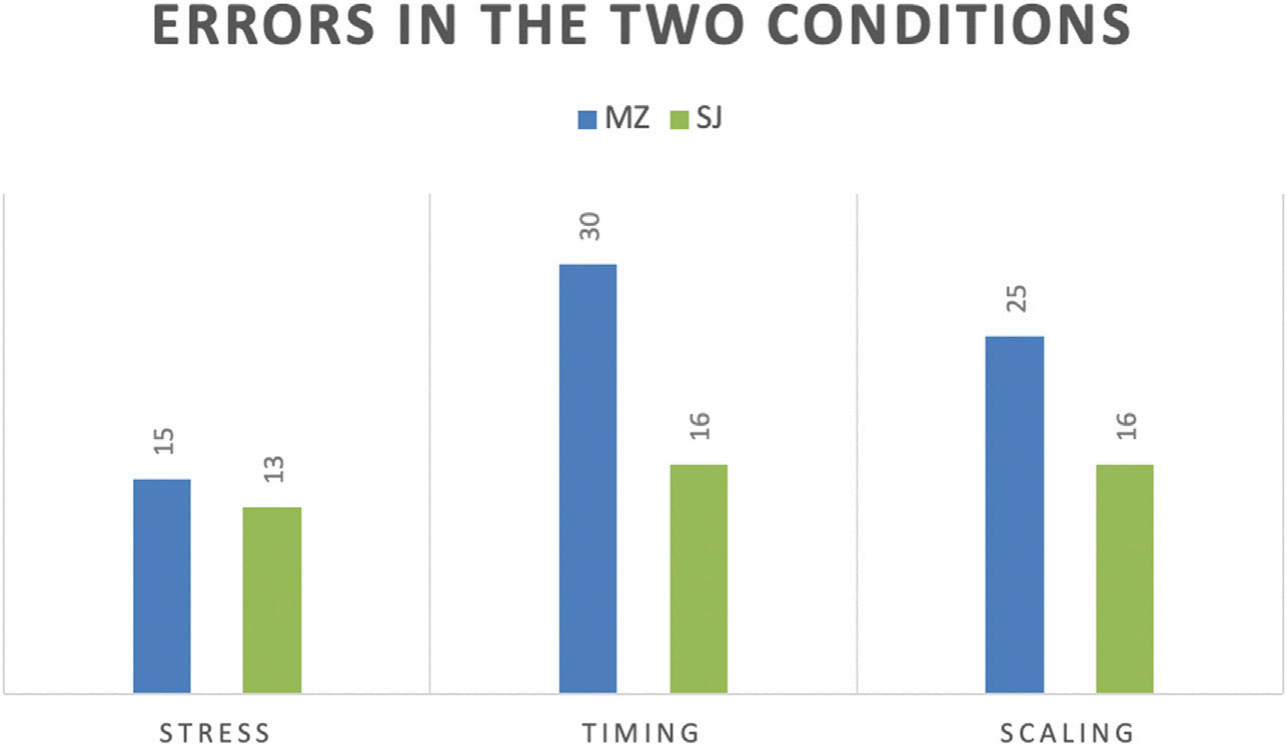
Distribution of the errors made by students in the two conditions.

Table 2 summarizes the errors made in the two conditions, and Figure 3 illustrates their distribution by condition. We carried out a Chi Square test on the data and find a very significant difference between the two conditions on all errors (*X* ^*2*^ (1, *N* = 40) = 7.9858, *p* = 0.004715, η^2^ = 0.19). Furthermore, the differences in errors of timing are very significant (*X* ^*2*^ (1, *N* = 40) = 6.9095, *p* = 0.008574, η^2^ = 0.17), and the difference between the scaling errors approaches significance (*X* ^*2*^ (1, *N* = 40) = 3.0009, *p* = 0.083217, η^2^ = 0.08). Just regarding the stress placement, the difference is non-significant.

**TABLE 2:** Stress, timing and scaling errors made by students in the Steve Jobs (SJ) and the Mark Zuckerberg (MZ) conditions.

Condition\Error	Stress	Timing	Scaling	Total
SJ	13 (21.7%)	16 (26.7%)	16 (26.7%)	45 (25%)
MZ	15 (25%)	30 (50%)	25 (41.7%)	70 (38.9%)

**FIGURE 4:**
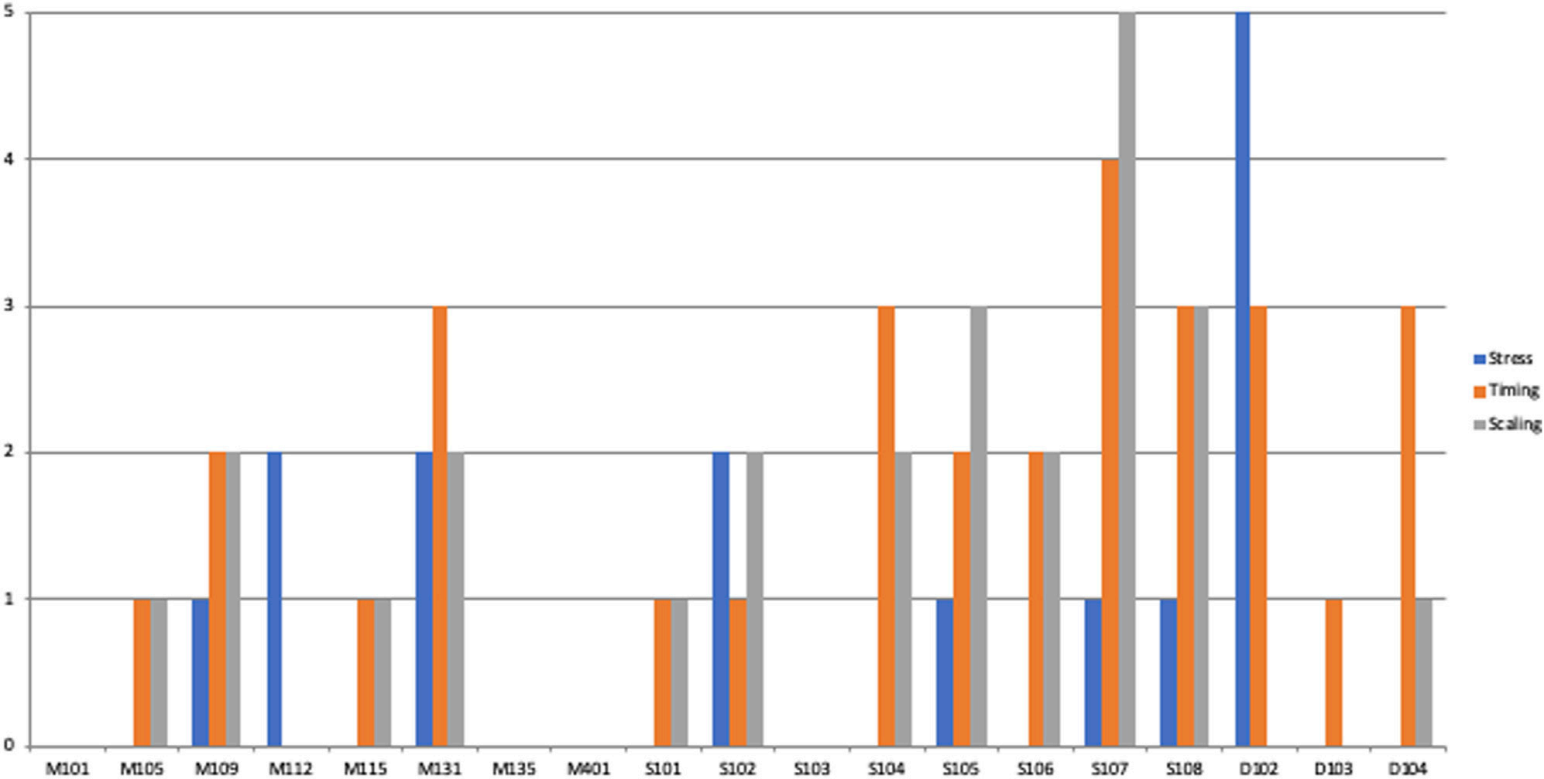
Errors made by the individual participants in the Mark Zuckerberg-condition.

**FIGURE 5:**
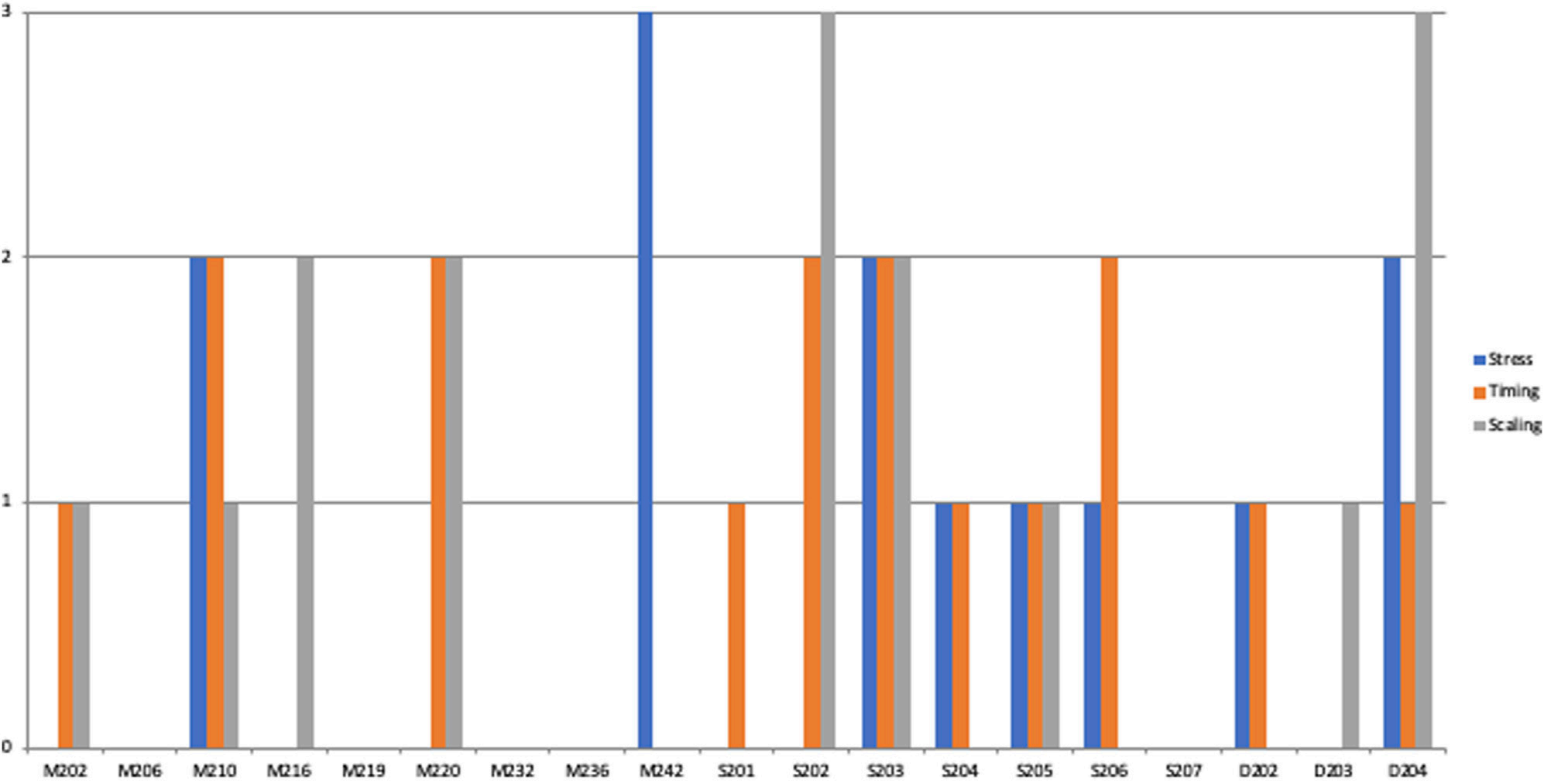
Distribution of errors by participants in the Steve Jobs-condition.

Figures 4, 5 illustrate the distributions of the different errors in the two conditions by participant; they show that the different speaking styles affected a large number of participants, and that the effect is not due to a few outliers.

## Discussion

The results suggest that the task chosen, to produce English questions with the appropriate prosodic features, was sufficiently difficult, and that even in the condition in which the robot was presenting the instructions with speech characteristics based on Steve Jobs’ speech, students still made many errors, including scaling errors that concern the direction of the pitch movement (up or down). Similarly, the timing and placement of stress was challenging. The task was thus adequately difficult but not impossible for the students, and to acquire native-like competence with respect to intonation has been identified as challenging across foreign language teaching research (e.g., Levis 2018). We can conclude that the results were not influenced by potential floor or ceiling effects.

The results obtained furthermore indicate that a teacher’s speaking style has a small, but consistent impact on students’ objective performance, even if that teacher is a robot. Thus, there is evidence that this part of a teacher’s personality significantly influences how well students perform on a given task. While it is still unclear to what extent charisma can be learnt (cf. Abelin 2018), robots can take over the role of providing charismatic instructions in teaching materials.

We suspect that the effect of speaking style on student performance is due to the pragmatic and cognitive effects of charismatic speech in terms of perceived competence, self-confidence and passion. The speech characteristics chosen can be related empirically to these three personality traits, which are likely to cause the effects observed. Specifically, speaking rate and silent pause duration are mostly related to competence (cf. Niebuhr and Michalsky 2019), whereas pitch range and emphatic-accent frequency are related to passion and a higher level of arousal. These in turn can lead to heightened attention and memory in the listener (cf. Niebuhr 2021b). For instance, conveying competence creates trust (“the speaker can do that”), conveying self-confidence creates motivation (“I can do that, too”), and conveying passion creates inspiration and commitment (“I want to do that, too”). This is, we suspect, the reason why a charismatic teacher increases students’ performance.

The domain we investigated concerns pronunciation, and thus we have no results on other areas of foreign language learning, such as grammar, vocabulary or interaction. Furthermore, our study focused on adult language learners, and hence the effects may be different for children. Currently, we cannot see a reason why our results should not carry over to other subject areas and other populations, but future work is needed to confirm a general effect.

Another possible limitation may be that the robot only provides the general instructions concerning the different steps involved in the task, so that there is no “teaching” involved in the sense of clarifying contents for a learner. However, we believe that the results are therefore all the more interesting just because the role of the robot as a teacher is so small; if this short introduction to the task already affects the students’ performance, then the robot’s speech characteristics are likely to affect student performance even more so with a greater involvement of the robot as teacher.

In spite of these potential limitations, we can conclude that robots may serve well as instructors in language teaching, but that their success depends at least to some extent on the speaking style used. Given that most available text-to-speech systems do not provide speaking styles that exhibit characteristics identified as charismatic in previous work, and instead are characterized by features that range low on the charismatic side (see In and Han 2015), this finding draws attention to the need for more adequate speech synthesis for human-robot interaction. Furthermore, our findings suggest that some of the negative findings on robot tutors might actually be due to the respective robot’s speaking style - future work will have to shed light on the magnitude of this effect. Finally, the results suggest that speaking style contributes significantly to (robot) teacher personality and student performance.

## Data Availability

The raw data supporting the conclusions of this article will be made available by the authors, without undue reservation.
